# Stability Analysis of Distributed Order Fractional Chen System

**DOI:** 10.1155/2013/645080

**Published:** 2013-12-29

**Authors:** H. Aminikhah, A. Refahi Sheikhani, H. Rezazadeh

**Affiliations:** ^1^Department of Applied Mathematics, School of Mathematical Sciences, University of Guilan, P.O. Box 1914, Rasht, Iran; ^2^Department of Applied Mathematics, Faculty of Mathematical Sciences, Islamic Azad University, Lahijan Branch, P.O. Box 1616, Lahijan, Iran

## Abstract

We first investigate sufficient and necessary conditions of stability of nonlinear distributed order fractional system and then we generalize the integer-order Chen system into the distributed order fractional domain. Based on the asymptotic stability theory of nonlinear distributed order fractional systems, the stability of distributed order fractional Chen system is discussed. In addition, we have found that chaos exists in the double fractional order Chen system. Numerical solutions are used to verify the analytical results.

## 1. Introduction

The history of fractional calculus is more than three centuries old, yet only in the past 20 years has the field received much attention and interest. The reader may refer to [1, 2]. The generalization of dynamical equations using fractional derivatives proved to be useful and more accurate in mathematical modeling related to many interdisciplinary areas. Applications of fractional calculus and fractional-order differential equations include dielectric relaxation phenomena in polymeric materials [3], transport of passive tracers carried by fluid flow in a porous medium in groundwater hydrology [4], transport dynamics in systems governed by anomalous diffusion [5, 6], long-time memory in financial time series [7], and so on [8, 9]. Stability analysis and control systems are two of the most important problems such that, in 1996, Matignon [10] firstly studied stability of *n*-dimensional linear fractional systems from a point of view of control. Since then, many researchers have completed further studies on the stability of linear fractional differential systems [11–14]. For the nonlinear fractional differential systems, the stability analysis is much more difficult and only a few are available. Some authors [15, 16] studied the nonlinear fractional differential system.

Nonlinear systems are very interesting to engineers, physicists, and mathematicians because most real physical systems are inherently nonlinear in nature. Nonlinear dynamical systems can exhibit completely an unpredictable behavior, the so-called deterministic chaos. Chaos is an important dynamical phenomenon that has been extensively studied and developed by scientists and engineers in the last decades in various fields such as physical [17], chemical [18], and ecological systems [19]. Since chaos is useful and has potential applications in many technological disciplines, the discovery and the creation of chaos are important. In 1963, Lorenz found the first chaotic attractor in a three-dimensional autonomous system [20]. Later, several dynamical systems exhibiting chaos have been presented in various branches of science [21]. For example, in 1999, Chen and Ueta found another simple three-dimensional autonomous system, which is not topologically equivalent to Lorenz's system and which has a chaotic attractor [22, 23]. Similar to integer-order chaotic systems, the fractional order chaotic systems have also interested several researchers. I. Grigorenko and E. Grigorenko extended the study of this prototypical system to equations of fractional order but, unfortunately, the results presented in this paper are not correct [24]. In [25, 26], the chaotic behavior and its control in the fractional order Chen system are investigated. Also, chaotic behaviors have been found in the fractional order systems of Chuna [27], Rossler [28], Coullet [29], modified Van der Pol-Duffing [30], and Liu [31]. In addition, the fractional order Chen system has been studied with time-delay in [32].

The idea of fractional derivative of distributed order is stated by Caputo [33] and later developed by Caputo himself [34, 35], and Bagley and Torvik [36, 37]. Other researchers used this idea, and interesting reviews appeared to describe the related mathematical models of partial fractional differential equation of distributed order. For example, Diethelm and Ford [38] used a numerical technique along with its error analysis to solve the distributed order differential equation and analyze the physical phenomena and engineering problems; see [38] and references therein. Recently Saberi Najafi et al. [39, 40] studied stability analysis of distributed order fractional differential equations with respect to the nonnegative density function. The aim of the present work is twofold. First, we consider the stability of *n*-dimensional nonlinear distributed order fractional differential system with respect to the nonnegative density function and then we study the stability of distributed order fractional Chen system.

This paper is organized as follows. In the next section, we recall some basic definitions of the Caputo fractional derivative operator, systems with fractional derivatives of distributed order.  Section 3 contains the main definitions and theorems for checking the stability analysis of distributed order fractional system. In Section 4, we present the distributed order fractional Chen system and afterwards based on the stability theorem of distributed order fractional systems, the stability of the distributed order fractional Chen system is discussed. Finally, the numerical solution to illustrate the validity of the results is presented in Section 5.

## 2. Elementary Definitions

In this section, we consider the main definitions and properties of fractional derivative operators of single and distributed order.

The differential and integral operator in fractional calculus is denoted by _*a*_ *D*_*t*_^*α*^, where *a* and *t* are the bounds of the operation and *α* is the fractional order, which can be rational, irrational, or even complex. For simplicity and without loss of generality, in the following, we assume that *a* = 0 and *D*_*t*_^*α*^ = _0_ *D*_*t*_^*α*^. The continuous integrodifferential operator is defined as follows:

(1) $$\begin{array}{c} D_{t}^{\alpha }=\{\begin{array}{cc} \frac{d^{\alpha }}{{dt}^{\alpha }}, & \alpha >0,\\ 1, & \alpha =0,\\ \overset{t}{\underset{0}{\int }}{\left(d\tau \right)}^{-\alpha }‍, & \alpha <0. \end{array} \end{array}$$

There are many definitions of fractional derivatives of order *α* > 0 [1, 2], such as Grunwald-Letnikov's definition (GL), Riemann-Liouville's definition (RL), and Caputo's fractional derivative. The RL definition is given as

(2) DtαRLf(t)=1Γ(n−α)dndtn∫0t(t−τ)n−α−1f(τ)dτ,

where *n* is the first integer which is not less than *α*; that is, *n* − 1 < *α* < *n* and Γ(·) is a Gamma function. The Caputo fractional derivative of *f*(*t*) is defined as

(3) DCtαf(t)=1Γ(n−α)∫0t(t−τ)n−α−1f(n)(τ)dτ.

Finally, Grunwald-Letnikov definition is given by

(4) DGLtαf(t)=lim⁡h→0⁡h−α∑i=0[(t−a)/h](−1)i(αi)f(t−jh).

Fortunately, the Laplace transform of the Caputo fractional derivative satisfies

(5) ℒ{Dctαf(t)}=sαℒ{f(t)}−∑k=0m−1f(k)(0+)sα−1−k,

where *m* − 1 < *α* ≤ *m* and *s* is the Laplace variable. The Laplace transform of Caputo fractional derivative requires the knowledge of the initial values of the function and its integer derivatives of order *k* = 1,2,…, *m* − 1. When *α* ∈ (0,1], is given by

(6) ℒ{Dctαf(t)}=sαℒ{f(t)}−f(0+)sα−1.

When the fractional calculus operators act on *f*(*t*), and we integrate _*a*_ *D*_*t*_^*α*^ *f*(*t*) with respect to the order, then distributed order fractional differential equations can be obtained. In this brief, the following distributed order fractional differential operator notation is adopted:

(7) Dctb(α)f(t)=∫m−1mb(α)Dctαf(t)dα, m−1≤α≤m,  m∈ℕ,

where the derivative ^*c*^ *D*_*t*_^*α*^ is taken to be a fractional derivative of Caputo type of order *α* with respect to the nonnegative density function *b*(*α*).

The idea of distributed order is stated by Caputo [33, 34]. Further the Laplace transform of the Caputo distributed order satisfies

(8) ℒ{Dctb(α)f(t)}=∫m−1mb(α)   ×[sαF(s)−∑k=0m−1f(k)(0+)sα−1−k]dα=B(s)F(s)−∑k=0m−11sk+1B(s)f(k)(0+),

where *F*(*s*) is the Laplace transform of *f*(*t*) and

(9) $$\begin{array}{c} B\left(s\right)=\overset{m}{\underset{m-1}{\int }}b\left(\alpha \right)s^{\alpha }d\alpha . \end{array}$$

A distributed order fractional equations can be defined by the following model:

(10) Dctb(α)x(t)=f(t,x(t)),x(0)=x0,

where *x*(*t*) ∈ ℝ and ^*c*^ *D*_*t*_^*b*(*α*)^ *x*(*t*) = ∫_0_^1^ *b*(*α*)^*c*^ *D*_*t*_^*α*^ *x*(*t*)*dα*.

In [38] the following results about the existence and uniqueness of solutions for (10) are further presented.

**Theorem 1**

Let the function *b* be absolutely integrable on the interval [0,1] and satisfy ∫_0_^1^ *b*(*α*)*s* ^*α*^ *dα* ≠ 0 for **R** **e**(*s*) > 0 and *f* ∈ **L** ^1^[0, *∞*) and *x* is such that ^*c*^ *D*_*t*_^*α*^ *x*(*t*) < **M** for *t* ∈ [0, *∞*] for every *α* ∈ [0,1]; then initial value problem (10) has a unique solution.

Furthermore, the above definition in one dimension can naturally be generalized to the case of multiple dimensions; that is, let *X*(*t*) = (*x*_1_(*t*), *x*_2_(*t*),…, *x*_*n*_(*t*))^*T*^ ∈ ℝ^*n*^ and *b*(*α*) = (*b*_1_(*α*), *b*_2_(*α*),…, *b*_*n*_(*α*))^*T*^, 0 < *α* < 1. The *n*-dimension distributed order fractional system is described as follows:

(11) Dctb(α)X(t)=∫01b(α)DctαX(t)dα=F(t,X(t)),

where

(12) Dctb(α)X(t) =(Dctb1(α)x1(t),Dctb2(α)x2(t),…,Dctbn(α)xn(t)),F(t,X(t))=(f1(t,x1(t),x2(t),…,xn(t))f2(t,x1(t),x2(t),…,xn(t))⋮fn(t,x1(t),x2(t),…,xn(t))).

The result of Theorem 1 can be easily generalized to the initial value problem of (11).

## 3. Stability Analysis of Distributed Order Fractional Systems

In this section, we generalize the main stability properties for systems of distributed order fractional. The linear distributed order fractional systems are expressed as

(13) Dctb(α)X(t)=AX(t),X(0)=X0,

where *X*(*t*) = (*x*_1_(*t*), *x*_2_(*t*),…, *x*_*n*_(*t*))^*T*^ ∈ ℝ^*n*^, the matrix *A* ∈ ℝ^*n*×*n*^, and *b*(*α*) = (*b*_1_(*α*), *b*_2_(*α*),…, *b*_*n*_(*α*))^*T*^, 0 < *α* < 1. Then Saberi Najafi et al. [39] have obtained the general solution of the distributed order fractional systems (13), which is written by

(14) X(t)=X(0)+1π∫0t∫0∞∫0∞e−rt+Aτ−ρcos⁡⁡(πγ)×sin⁡(ρsin⁡(πγ))sin⁡AX(0)dr dτ dt,

where *B*(*s*) = *ρ*cos⁡⁡(*πγ*) + *iρ*sin⁡(*πγ*), *ρ* = |*B*(*s*)|, *γ* = (1/*π*)arg[*B*(*s*)], and *r* = *e* ^*iπ*^.

Now, we will recall some theorems and definitions about linear distributed order fractional equations and then we will show this theorem for nonlinear distributed order fractional equations as well.

**Theorem 2 (see [39])**

The distributed order fractional system of (13) is asymptotically stable if and only if all roots of *det*(*B*(*s*)*I* − *A*) = 0 have negative real parts.

**Definition 3 (see [39])**

The value of det⁡(*B*(*s*)*I* − *A*) = 0 is the characteristic function of the matrix *A* with respect to the distributed function *B*(*s*), where *B*(*s*) = ∫_0_^1^ *b*(*α*)*s* ^*α*^ *dα* is the distributed function with respect to the density function *b*(*α*) ≥ 0.

The inertia of a matrix is the triplet of the numbers of eigenvalues of *A* with positive, negative, and zero real parts. As pointed out in [39], authors have generalized the inertia concept for analyzing the stability of linear distributed order fractional systems.

**Definition 4**

The inertia of the system (13) is the triple

(15) $$\begin{array}{c} I_{n_{B(s)}}\left(A\right)=\left(\pi_{B(s)}\left(A\right),\upsilon_{B(s)}\left(A\right),\delta_{B(s)}\left(A\right)\right), \end{array}$$

where *π*_*B*(*s*)_(*A*), *υ*_*B*(*s*)_(*A*), and *δ*_*B*(*s*)_(*A*) are, respectively, the number of roots of det⁡(*B*(*s*)*I* − *A*) = 0 with positive, negative, and zero real parts, where *B*(*s*) = (*B*_1_(*s*), *B*_2_(*s*),…, *B*_*n*_(*s*))^*T*^ is the distributed function with respect to the density function *b*(*α*) = (*b*_1_(*α*), *b*_2_(*α*),…, *b*_*n*_(*α*))^*T*^.

**Theorem 5 (see [39])**

The linear distributed order fractional system (13) is asymptotically stable if and only if any of the following equivalent conditions holds:

1. *π*_*n*_*B*(*s*)__(*A*) = *δ*_*n*_*B*(*s*)__(*A*) = 0,
2. all roots *s* of the characteristic function of *A* with respect to *B*(*s*) = (*B*_1_(*s*), *B*_2_(*s*),…, *B*_*n*_(*s*))^*T*^ satisfy |*arg*(*s*)|>*π*/2.

Next, we will mainly discuss the stability of a nonlinear autonomous distributed order fractional system, which can be described by

(16) Dctb(α)X(t)=F(X(t)),

with the initial value *X*(0) = *X*_0_, where

(17) $$\begin{array}{c} F\left(X\left(t\right)\right)=\left(\begin{array}{c} f_{1}\left(x_{1}\left(t\right),x_{2}\left(t\right),\ldots ,x_{n}\left(t\right)\right)\\ f_{2}\left(x_{1}\left(t\right),x_{2}\left(t\right),\ldots ,x_{n}\left(t\right)\right)\\ \vdots \\ f_{n}\left(x_{1}\left(t\right),x_{2}\left(t\right),\ldots ,x_{n}\left(t\right)\right) \end{array}\right), \end{array}$$

*X*(*t*) = (*x*_1_(*t*), *x*_2_(*t*),…, *x*_*n*_(*t*))^*T*^ ∈ ℝ^*n*^, and *b*(*α*) = (*b*_1_(*α*), *b*_2_(*α*),…, *b*_*n*_(*α*))^*T*^, 0 < *α* < 1.

**Theorem 6**

Let $\hat{X}={\left({\hat{x}}_{1},{\hat{x}}_{2},\ldots ,{\hat{x}}_{n}\right)}^{T}$ be the equilibrium of system (16); that is, ${\;}^{c}D_{t}^{b(\alpha )}\hat{X}=F(\hat{X})=0$ and $J=(\partial F/\partial X){|}_{X=\hat{X}}$ is the Jacobian matrix at the point $\hat{X}$; then the point $\hat{X}$ is asymptotically stable if and only if all roots *s* of the characteristic function of **J** with respect to *B*(*s*) = (*B*_1_(*s*), *B*_2_(*s*),…, *B*_*n*_(*s*))^*T*^ satisfy |*arg*(*s*)|>*π*/2.

**Proof**

Let $\zeta (t)=X(t)-\hat{X}$, where *ζ*(*t*) = (*ζ*_1_(*t*), *ζ*_2_(*t*),…, *ζ*_*n*_(*t*)) is a small disturbance from a fixed point. Therefore

(18) Dctb(α)ζ(t)=Dctb(α)(X(t)−X^),

since Dctb(α)(X(t)-X^)=Dctb(α)X(t)-Dctb(α)X^ and Dctb(α)X^=0; thus, we have

(19) Dctb(α)ζ(t)=Dtb(α)cX(t)=F(X(t))=F(ζ(t)+X^)=F(X^)+Jζ(t)+higher  order  terms≈Jζ(t).

System (18) can be written as

(20) Dctb(α)ζ(t)≈Jζ(t),

with the initial value $\zeta (0)=X_{0}-\hat{X}$.

The analytical procedure of linearization is based on the fact that if the matrix **J** has no purely imaginary eigenvalues, then the trajectories of the nonlinear system in the neighborhood of the equilibrium point have the same form as the trajectories of the linear system [41]. Hence, by applying Theorem 2, the linear system (20) is asymptotically stable if and only if all roots *s* of the characteristic function of **J** with respect to *B*(*s*) = (*B*_1_(*s*), *B*_2_(*s*),…, *B*_*n*_(*s*))^*T*^ satisfy |arg(*s*)|>*π*/2, which implies that the equilibrium $\hat{X}$ of the nonlinear distributed order fractional system (16) is as asymptotically stable.

**Remark 7**

The nonlinear distributed order fractional system (16) in the point $\hat{X}$ is asymptotically stable if and only if *π*_*n*_*B*(*s*)__(*J*) = *δ*_*n*_*B*(*s*)__(*J*) = 0.

## 4. Distributed Order Fractional Chen System

**Figure 1:**
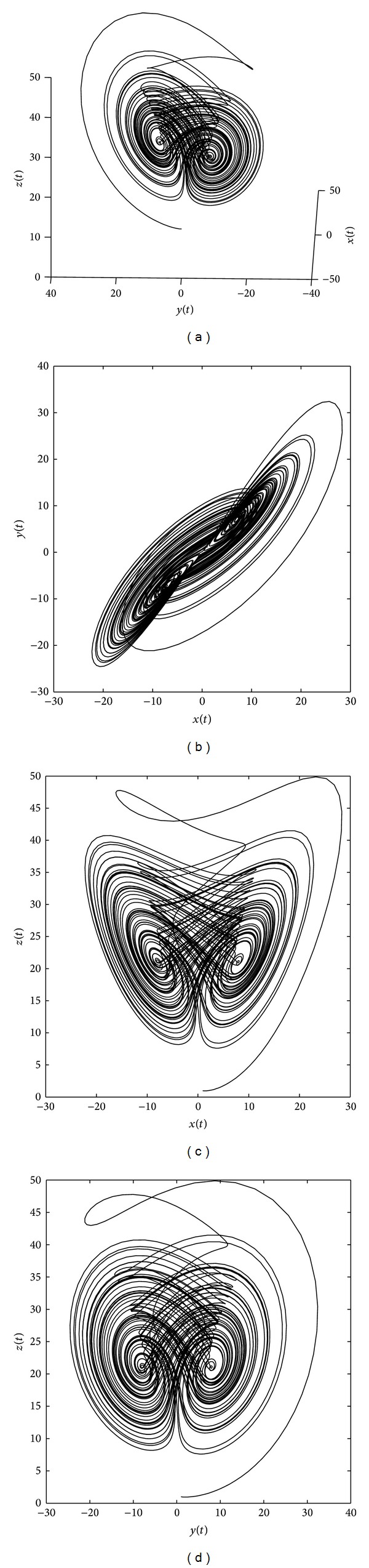
Chaotic attractor of the integer-order Chen system (21) with (*a*, *b*, *c*) = (35, 3, 28).

The Chen system is described by the following nonlinear differential equations on **R** ^3^ [22, 23]:

(21) x˙(t)=a(y(t)−x(t)),y˙(t)=(c−a)x(t)−x(t)z(t)+cy(t),z˙(t)=x(t)y(t)−bz(t),

where *x*, *y*, and *z* are the state variables and *a*, *b*, and *c* are three system parameters. The above system has a chaotic attractor when *a* = 35, *b* = 3, and *c* = 28 as shown in Figure 1. The corresponding distributed order fractional Chen system (21) can be written in the form

(22) Dtb1(α)cx(t) =a(y(t)−x(t)),Dtb2(α)cy(t) =(c−a)x(t)−x(t)z(t)+cy(t),Dtb3(α)cz(t) =x(t)y(t)−bz(t),

where *b*_*i*_(*α*) for *i* = 1,2, 3 denote the nonnegative density function of order *α* ∈ (0,1]. As a generalization of nonlinear fractional order differential equation into nonlinear distributed order fractional differential equation, the linearized form of the system (22) at the equilibrium points ${\hat{x}}_{1}=(0,0,0)$, ${\hat{x}}_{2}=(\sqrt{b(2c-a)},\sqrt{b(2c-a)},2c-a)$, and ${\hat{x}}_{3}=(-\sqrt{b(2c-a)},-\sqrt{b(2c-a)},2c-a)$, that is, Dctb1(α)x^(t)=F(x^)=0, can be written in the form

(23) Dctb(α)X(t)=JX(t),

where *X*(*t*) = (*x*(*t*), *y*(*t*), *z*(*t*))^*T*^, *b*(*α*) = (*b*_1_(*α*), *b*_2_(*α*), *b*_3_(*α*))^*T*^, 0 < *α* ≤ 1, and $J=(\partial F/\partial X){|}_{X={\hat{x}}_{i}}$ for *i* = 1,2, 3. The Jacobian matrix of distributed order fractional Chen system (22) at the equilibrium point X* = (x*, y*, z*) is given by

(24) $$\begin{array}{c} J=\left[\begin{array}{ccc} -a & a & 0\\ c-a-z^{*} & c & -x^{*}\\ y^{*} & x^{*} & -b \end{array}\right]. \end{array}$$

**Remark 8**

If *b*_*i*_(*α*) = *δ*(*α* − *q*_*i*_), where 0 < *q*_*i*_ ≤ 1 for *i* = 1,2, 3 and *δ*(*α*) is the Dirac delta function, then, we have the following fractional incommensurate-order Chen system [25]:

(25) Dtα1cx(t) =a(y(t)−x(t)),Dtα2cy(t) =(c−a)x(t)−x(t)z(t)+cy(t),Dtα3cz(t) =x(t)y(t)−bz(t).

Based upon Theorem 6, the stability of the distributed order fractional Chen system can be reached with ease. For analyzing the stability of the distributed order fractional Chen system, we compute *I*_*n*_*B*(*s*)__(**J**) in the case that the density function varies. The results are shown in Table 1 for some parameters *a*, *b*, and *c*.

## 5. Numerical Methods

As pointed out in [38], distributed order fractional differential equations may be regarded as a generalization of single-term fractional differential equations

(26) Dctαx(t)=f(t,x(t))

or multiterm fractional differential equations

(27) ∑i=1nγiDctαix(t)=f(t,x(t)), 0<α1<α2<⋯<αn.

Therefore, in this section, numerical method to solving of (26) and (27) is presented. The approximate Grunwald-Letnikov's definition is given below, where the step size of *h* is assumed to be very small [2, 42, 43]

(28) Dctkαy(t)≈h−α∑i=0kci(α)y(tk−i),

where *t*_*k*_ = *kh*  (*k* = 0,1,…) and *c*_*i*_^(*α*)^  (*i* = 0,1,…) are binomial coefficients, which can be computed as [43]

(29) $$\begin{array}{c} c_{0}^{\left(\alpha \right)}=1,\;\;c_{i}^{\left(\alpha \right)}=\left(1-\frac{1+\alpha }{i}\right)c_{i-1}^{\left(\alpha \right)}. \end{array}$$

Then, general numerical solution of (26) and (27) can be expressed as

(30) h−α∑i=0kci(α)x(tk−i)=f(t,x(t)),γ1h−α1∑i=0kci(α1)x(tk−i)+⋯+γnh−αn∑i=0kci(αn)x(tk−i)  =f(t,x(t)),

where *c*_*i*_^(*α*_*j*_)^ for *j* = 1,…, *n* are binomial coefficients calculated according to (29). Equations in (30) can be rewritten as the following forms:

(31) x(tk)=f(x(tk−1),tk−1)hα−∑i=1kci(α)x(tk−i),x(tk) =1∑j=1najh−αj  ×[f(x(tk−1),tk−1)−∑j=1nγjh−αj∑i=1kci(αj)x(tk−i)].

Based on the previous algorithm, we can obtain the numerical solution of the fractional differential equations (26) and multiterm fractional differential equations (27), where *k* = 1,2,…, *N* for *N* = *T*/*h* and where *T* is the total time of the calculation.

To verify the efficiency of the obtained results in Table 1, the numerical solution for the distributed order fractional Chen system has been computed. In the following calculations, let *T* = 10, *h* = 0.005 with the initial conditions (0.2, −0.5,0.2).

**Figure 2:**
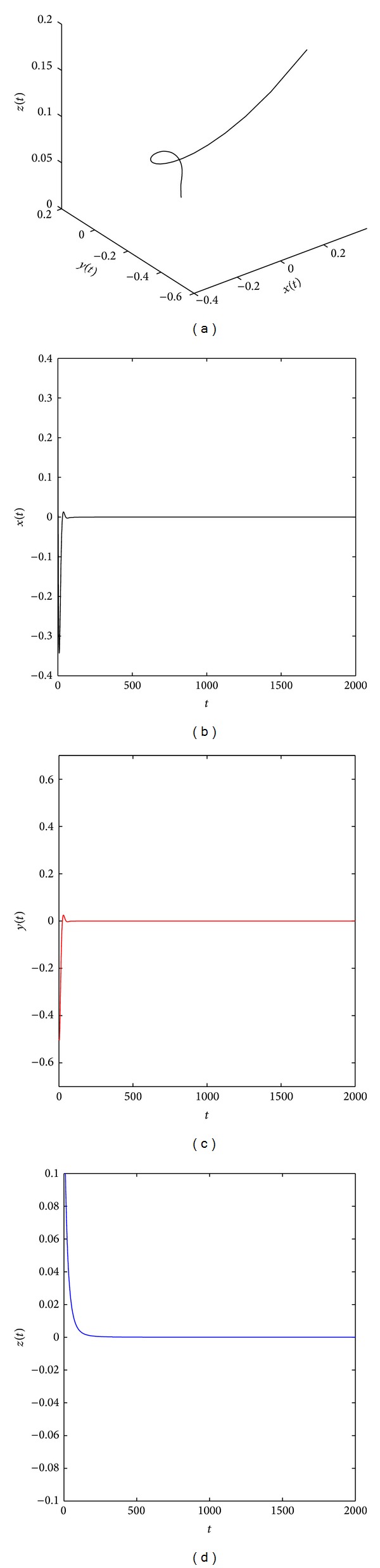
The equilibrium point ${\hat{x}}_{1}$ of the distributed order fractional Chen system (22) with (*q*_1_, *q*_2_, *q*_3_) = (0.95, 0.9, 0.9) and (*a*, *b*, *c*) = (35, 6, 11) is asymptotically stable.

**Figure 3:**
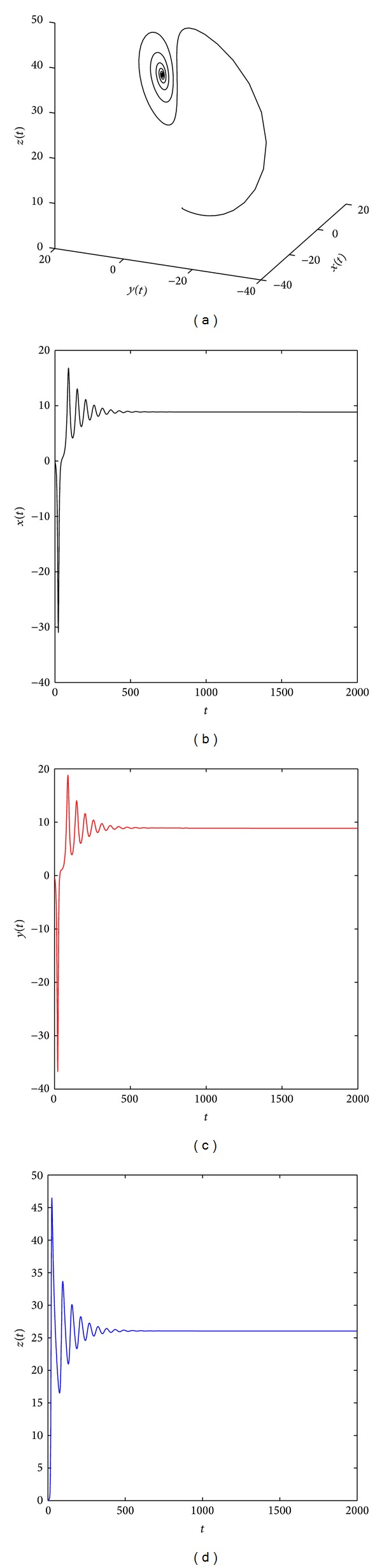
The equilibrium point ${\hat{x}}_{2}$ of the distributed order fractional Chen system (22) with (*q*_1_, *q*_2_, *q*_3_) = (0.7, 0.8, 0.9) and (*a*, *b*, *c*) = (30, 3, 28) is asymptotically stable.

**Figure 4:**
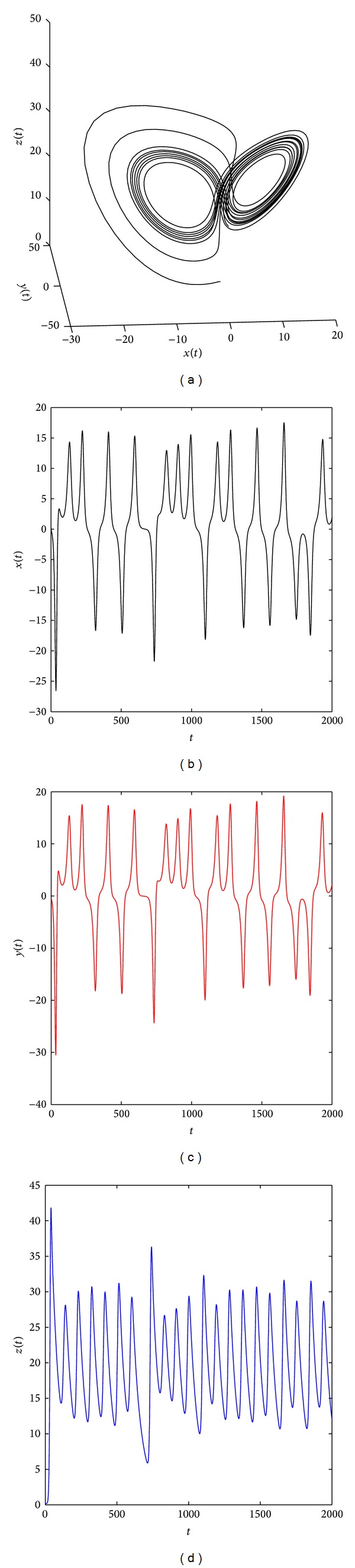
Chaotic attractor of the distributed order fractional Chen system (22) with (*q*_1_, *q*_2_, *q*_3_) = (0.85, 0.9, 0.95) and (*a*, *b*, *c*) = (35, 3, 28).

**Figure 5:**
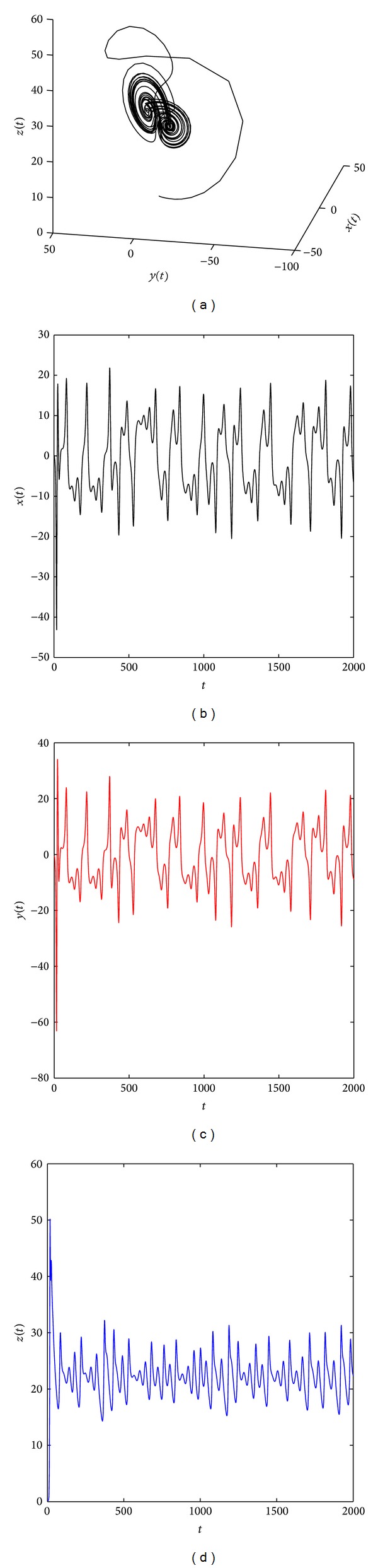
Chaotic attractor of the distributed order fractional Chen system (22) with (*q*_11_, *q*_12_, *q*_21_, *q*_22_, *q*_31_, *q*_32_) = (0.85, 0.3, 0.85, 0.3, 0.85, 0.3) and (*a*, *b*, *c*) = (35, 3, 29).

**Figure 6:**
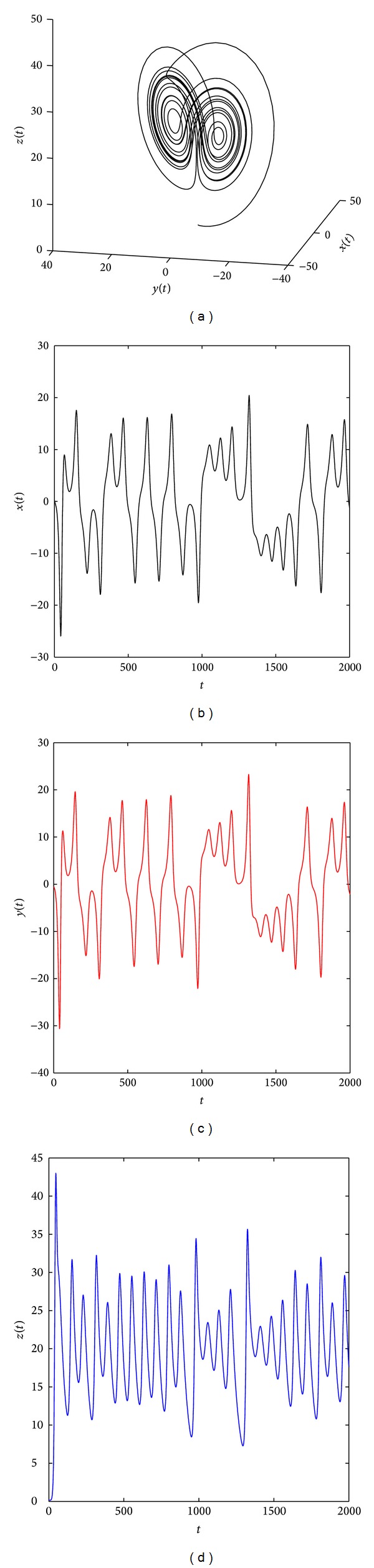
Chaotic attractor of the distributed order fractional Chen system (22) with (*q*_11_, *q*_12_, *q*_21_, *q*_22_, *q*_31_, *q*_32_) = (0.95, 0.1, 0.95, 0.1, 0.95, 0.1) and (*a*, *b*, *c*) = (35, 3, 28).

**Figure 7:**
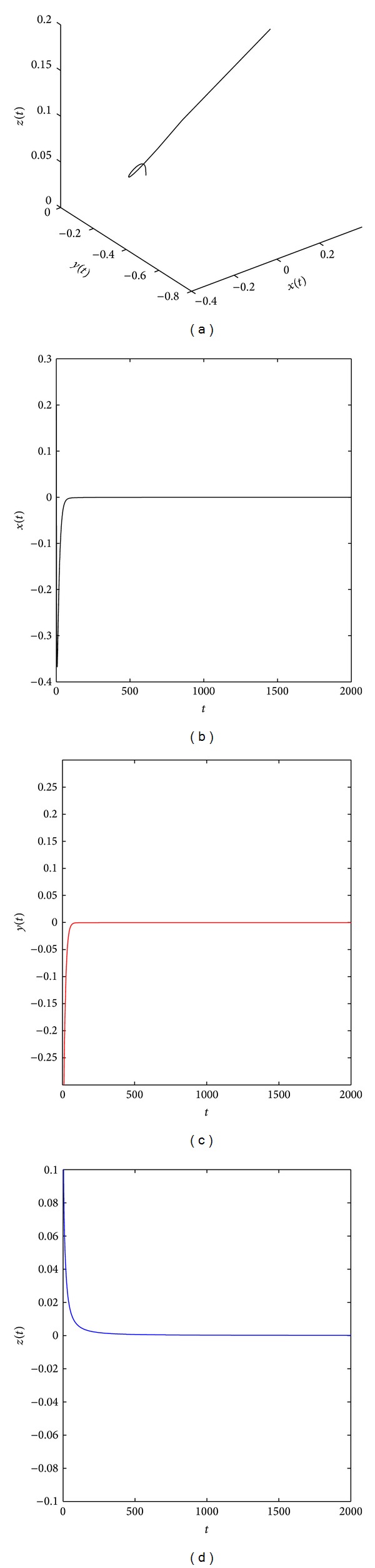
The equilibrium point ${\hat{x}}_{1}$ of the distributed order fractional Chen system (22) with (*q*_11_, *q*_12_, *q*_21_, *q*_22_, *q*_31_, *q*_32_) = (0.25, 0.85, 0.25, 0.95, 0.25, 0.75) and (*a*, *b*, *c*) = (50, 3, 20) is asymptotically stable.

Figure 2 shows that the system (22) with parameters (*a*, *b*, *c*) = (35,6, 11) and (*q*_1_, *q*_2_, *q*_3_) = (0.95,0.9,0.9) is asymptotically stable in the equilibrium ${\hat{x}}_{1}$.  Figure 3 demonstrates that the system (22) with parameters (*a*, *b*, *c*) = (30,3, 28) and (*q*_1_, *q*_2_, *q*_3_) = (0.7,0.8,0.9) is asymptotically stable in the equilibrium ${\hat{x}}_{2}$. From Figure 4, we can see that for the distributed order Chen system (21) with parameters (*a*, *b*, *c*) = (35,3, 28) and (*q*_1_, *q*_2_, *q*_3_) = (0.85,0.9,0.95) is chaotic.  Figure 5 demonstrates that the system (22) with parameters (*a*, *b*, *c*) = (35,3, 29) and (*q*_11_, *q*_12_, *q*_21_, *q*_22_, *q*_31_, *q*_32_) = (0.85,0.3,0.85,0.3,0.85,0.3) has chaotic attractor.  Figure 6 shows the chaotic attractor for the distributed order Chen system (22) for parameters (*a*, *b*, *c*) = (35,3, 28) and (*q*_11_, *q*_12_, *q*_21_, *q*_22_, *q*_31_, *q*_32_) = (0.95,0.1,0.95,0.1,0.95,0.1). From Figure 7, we can see that the system (22) is asymptotically stable in the equilibrium ${\hat{x}}_{1}$ with parameters (*a*, *b*, *c*) = (50,3, 20) and (*q*_11_, *q*_12_, *q*_21_, *q*_22_, *q*_31_, *q*_32_) = (0.25,0.85,0.25,0.95,0.25,0.75).

## 6. Conclusion

In this paper, we introduced the nonlinear distributed order fractional differential equations with respect to a nonnegative density function; hence the asymptotical stability for such systems has been investigated. In addition, we presented the distributed order fractional Chen system and then in two special cases the stability for the distributed order fractional Chen is discussed. Numerical solutions were coincident with results of Table 1 described in Section 4. All numerical results are obtained using MATLAB 7.8.

## Figures and Tables

**Table 1:** Stability analysis of system (22) for various density functions.

*b*_*i*_(*α*) = *δ*(*α* − *q*_*i*_) *i* = 1,2, 3			*b*_*i*_(*α*) = *δ*(*α* − *q*_1*i*_) + *δ*(*α* − *q*_2*i*_) *i* = 1,2, 3		
(*a*, *b*, *c*)	*q* = (*q*_1_, *q*_2_, *q*_3_)	*I*_*n*_*B*(*s*)__ (**J**)	(*a*, *b*, *c*)	(q11,q12)(q21,q22)(q31,q32)	*I*_*n*_*B*(*s*)__ (**J**)

(35,6, 11)	(0.95,0.9,0.9)	x^1→(0,2,0)x^2=x^3→(1,2,0)	(35,3, 29)	(0.85,0.3)(0.65,0.3)(0.95,0.3)	x^1→(1,0,0)x^2=x^3→(2,0,0)

(30,3, 28)	(0.7,0.8,0.9)	x^1→(1,0,0)x^2→(0,2,0)	(35,3, 28)	(0.95,0.1)(0.95,0.1)(0.95,0.1)	x^1→(1,0,0)x^2=x^3→(20,18,0)

(35,3, 28)	(0.85,0.9,0.95)	x^1→(1,0,0)x^2=x^3→(2,0,0)	(50,3, 20)	(0.25,0.85)(0.25,0.95)(0.25,0.75)	x^1→(0,4,0)x^2x^3→(1,0,0)
